# Potential Application of the *Oryza sativa* Monodehydroascorbate Reductase Gene (*OsMDHAR*) to Improve the Stress Tolerance and Fermentative Capacity of *Saccharomyces cerevisiae*

**DOI:** 10.1371/journal.pone.0158841

**Published:** 2016-07-08

**Authors:** Il-Sup Kim, Young-Saeng Kim, Yul-Ho Kim, Ae-Kyung Park, Han-Woo Kim, Jun-Hyuk Lee, Ho-Sung Yoon

**Affiliations:** 1 School of Life Sciences, BK21 Plus KNU Creative BioResearch Group, Kyungpook National University, Daegu, Republic of Korea; 2 Division of Biological Sciences, University of California San Diego, La Jolla, California, United States of America; 3 Highland Agriculture Research Institute, National Institute of Crop Science, Rural Development Administration, Pyeongchang, Republic of Korea; 4 Division of Polar Life Sciences, Korea Polar Research Institute, Incheon, Republic of Korea; CNR, ITALY

## Abstract

Monodehydroascorbate reductase (MDHAR; EC 1.6.5.4) is an important enzyme for ascorbate recycling. To examine whether heterologous expression of *MDHAR* from *Oryza sativa* (*OsMDHAR*) can prevent the deleterious effects of unfavorable growth conditions, we constructed a transgenic yeast strain harboring a recombinant plasmid carrying *OsMDHAR* (p426GPD::OsMDHAR). *OsMDHAR*-expressing yeast cells displayed enhanced tolerance to hydrogen peroxide by maintaining redox homoeostasis, proteostasis, and the ascorbate (AsA)-like pool following the accumulation of antioxidant enzymes and molecules, metabolic enzymes, and molecular chaperones and their cofactors, compared to wild-type (WT) cells carrying vector alone. The addition of exogenous AsA or its analogue isoascorbic acid increased the viability of WT and *ara2Δ* cells under oxidative stress. Furthermore, the survival of *OsMDHAR*-expressing cells was greater than that of WT cells when cells at mid-log growth phase were exposed to high concentrations of ethanol. High *OsMDHAR* expression also improved the fermentative capacity of the yeast during glucose-based batch fermentation at a standard cultivation temperature (30°C). The alcohol yield of *OsMDHAR*-expressing transgenic yeast during fermentation was approximately 25% (0.18 g·g^-1^) higher than that of WT yeast. Accordingly, *OsMDHAR*-expressing transgenic yeast showed prolonged survival during the environmental stresses produced during fermentation. These results suggest that heterologous *OsMDHAR* expression increases tolerance to reactive oxygen species-induced oxidative stress by improving cellular redox homeostasis and improves survival during fermentation, which enhances fermentative capacity.

## Introduction

In aerobic organisms, oxygen is a double-edged sword; it is absolutely required for normal metabolic growth, yet continuous exposure to oxygen can cause cellular damage. This is because molecular oxygen is continually reduced within cells to reactive oxygen species (ROS), such as superoxide anions (O_2_*^−^), hydrogen peroxide (H_2_O_2_), and hydroxyl radicals (OH*). In addition, abiotic and biotic stimuli can lead to further ROS accumulation [1]. The produced ROS react readily with cellular components to generate acute or chronic damage that is sufficient to cause cell death, aging, and various disease [2]. Redox balance is maintained via the constitutive action of various antioxidant mechanisms that scavenge ROS, and both enzymatic and non-enzymatic processes can neutralize ROS [3].

Ascorbate (AsA)/D-erythroascorbate and glutathione are important antioxidants that are maintained in their reduced forms by enzymes in the AsA-glutathione cycle in higher eukaryotes, especially plants. The enzymes in this cycle, including AsA peroxidase (APX), monodehydroascorbate reductase (MDHAR), dehydroascorbate reductase (DHAR), and glutathione reductase (GR), are considered critical for minimizing and/or protecting cells from ROS induced by abiotic stressors [4]. MDHAR belongs to the flavoprotein family of pyridine nucleotide-disulfide oxidoreductases, which includes thioredoxin reductase (Trr), lipoamide dehydrogenase, GR, and mercuric ion reductase, and it catalyzes the reduction of monodehydroascorbate (MDHA) and MDHA radicals to AsA using NAD(P)H as an electron donor [5]. Therefore, MDHAR plays an important role in maintaining the pool of AsA derived from MDHA. MDHAR is found in many eukaryotes, including cucumbers, potatoes, soybean root nodules, and rot fungus [6], and it is localized in chloroplasts, mitochondria, peroxisomes, and the cytosol in plants [7], and in microsomes, mitochondria, the Golgi apparatus, and erythrocytes in animals [8]. MDHAR cDNAs have been cloned from the following plants: *Brassia rapa*, cucumber, pea leaves, tomatoes, rice, *Arabidopsis thaliana*, and leaf mustard [9–12]. The MDHAR amino acid sequence is unique and it shares limited identity with other flavin oxidoreductases in eukaryotes [5]. Transcriptional activation of the *MDHAR* gene has been reported under some environmental stresses, including oxidative stress such as H_2_O_2_, paraquat (PQ), and salicylic acid in *B*. *rapa* [13]; strong illumination in wheat leaves [14]; SO_2_ and O_3_ in conifer needles [15]; drought in grasses [16]; and internode rubbing in tomato plants [17].

*Saccharomyces cerevisiae* is a eukaryotic unicellular microfungus that is widely distributed in natural environments and in association with various insects, animals, and plants. It plays a critical role in food chains and the carbon, nutrient cycles such as nitrogen and sulfur, and is used to produce food, beverages, chemicals, pharmaceuticals, biocontrol agents, and industrial enzymes as well as in agriculture [18]. *S*. *cerevisiae* is the main yeast responsible for biomass-based alcoholic fermentation using sugars derived from rice, wheat, barley, corn, and grape in a commercially important agriculture sector [19]. During fermentation, yeast cells are dynamically challenged by mixed and interrelated stressors, especially ethanol [20]. Increased ethanol concentration stresses the cells and has an impact on fermentation quality and yield. Furthermore, ethanol production is dependent on the ability of the yeast to adapt to sudden or continuous changes during fermentation [21]. Specific properties of yeast can be altered by genetic improvement through numerous methodologies, including sexual breeding, parasexual hybridization (known as genome shuffling), random mutagenesis, genetic engineering (such as single chromosomal transfer), and transformation using recombinant tools. Using transgenic biotechnology, yeast are engineered to express foreign genes for the generation of products of industrial interest, such as bioethanol and secondary metabolites [22,23]. Because of their academic importance, studies of stress tolerance in yeast are of fundamental scientific importance. Stress intolerance can be overcome through the development of stress- and ethanol-tolerant yeast strains via effective engineering of relevant genes.

Unlike in plants, little is known about the stress response involving MDHAR in yeast. In this study, a cDNA encoding the cytosolic *MDHAR* from the rice plant *Oryza sativa* (*OsMDHAR*) was cloned, and its function was analyzed in a genetically modified *S*. *cerevisiae* strain. Herein, we report that heterologous *OsMDHAR* expression improves tolerance to ROS-induced oxidative stress and fermentative capacity in *S*. *cerevisiae*. Furthermore, these findings improve our understanding of the development of ethanol-tolerant yeast strains and demonstrate the potential application of transgenic yeast in the fermentation field for industrial bioethanol production using glucose-based biomass.

## Materials and Methods

### Construction of *OsMDHAR*-expressing transgenic yeast

Total RNA was isolated from the leaves of 4-week-old *O*. *sativa* seedlings, and the cDNA was synthesized by reverse transcription-polymerase chain reaction (RT-PCR). The *OsMDHAR* coding region was amplified from the cDNA by PCR using *Taq* and *Pfu* polymerases (Roche, Basel, Switzerland). The reaction conditions were as follows: initial denaturation at 94°C for 3 min, followed by 30 cycles of 94°C for 30 s, 56°C for 30 s, and 72°C for 2 min, and a final extension at 72°C for 7 min. The primer set used for PCR cloning of the *OsMDHAR* gene is shown in S1 Table. The PCR product was ligated into the pCR2.1^®^ TOPO-TA cloning vector (Invitrogen, Carlsbad, CA, USA). After sequence confirmation and digestion with *Eco*RI, the DNA fragment containing the *OsMDHAR* gene was ligated into the yeast expression vector p426GPD (Euroscarf, Frankfurt, Germany). The construct was transformed into *S*. *cerevisiae* BY4741 cells (S2 Table) and derivatives using the PEG/LiCl method [24]. Transformants were selected by plating cells on minimal agar medium (0.67% yeast nitrogen base without amino acids and with ammonium sulfate and 0.192% yeast synthetic drop-out medium lacking uracil, with 2% glucose, and 2% bacto agar) at 28°C for 3 days. Single colonies were restreaked, cultured under the same conditions, and then used for subsequent experiments.

### Semi-quantitative RT-PCR

Total RNA from mid-log phase yeast cells was obtained using the Total RNA Isolation Kit (Promega, Madison, WI, USA). RT-PCR was performed using Maxime RT-PCR Premix (iNtRON Biotechnology Inc., Seongnam, Korea) according to the manufacturer’s instructions. The oligonucleotide primers used were OsMDHAR-F and OsMDHAR-R (S1 Table). PCR amplicons were resolved in 1.5% agarose gels, stained with ethidium bromide, and photographed. The *PDA1* amplicon was used as a reference [25].

### Stress tolerance assay

To measure cell viability, cells (initial concentration adjusted to 1 × 10^6^ cells per mL) grown overnight at 28°C were inoculated into YPD medium (1% yeast extract, 2% peptone, and 2% dextrose) containing various concentrations of H_2_O_2_. The cells were cultured for 16 h at 28°C with shaking (160 rpm). After culture, the optical density at 600 nm (OD_600_) was measured. To monitor thegrowth kinetics, cells (starting concentration adjusted to 1 × 10^6^ cells per mL) were inoculated into YPD medium supplemented with 5 mM H_2_O_2_, and the OD_600_ was measured at 2-h intervals for the indicated times. For the spotting assay, mid-log phase cells (OD_600_ ≈ 2.0) were exposed to 20 mM H_2_O_2_ for 1 h at 28°C and serially diluted (10^0^ to 10^−4^). Then, a 5-μL aliquot of each dilution was spotted onto YPD agar plates and incubated for 2–3 days at 28°C. Additionally, to determine the stress sensitivity of mutant cells (*sod1Δ*, *tsa1Δ*, *por1Δ*, *por2Δ*, and *ara2Δ*; S2 Table), the OD_600_ of each mutant was adjusted to about 1.5, and then cells were exposed to 10 mM H_2_O_2_ for 1 h at 28°C, serially diluted, and then spotted onto YPD agar plates.

### Effect of exogenous antioxidants

Yeast cells in mid-log phase were treated with 10 mM AsA and 10 mM isoascorbate (IAA) for 1 h at 28°C, washed twice with cold phosphate-buffered saline (PBS) to remove residual antioxidants, and suspended in fresh YPD medium. Resuspended cells were exposed to 20 mM H_2_O_2_ for 1 h at 28°C with shaking, appropriately diluted, and spotted onto YPD agar plates. For growth kinetics, cells (1 × 10^6^ cells/mL) grown at 28°C overnight were inoculated into YPD medium containing 10 mM AsA and 10 mM IAA in the absence or presence of 5 mM H_2_O_2_. The OD_600_ was measured at 2-h intervals for the indicated time.

### Protein preparation

Mid-log phase cells were incubated in the absence or presence of 20 mM H_2_O_2_ for 1 h at 28°C and harvested by centrifugation at 4°C. Crude protein extracts were prepared using glass beads. Cells were washed three times with cold PBS and resuspended in lysis buffer (50 mM HEPES, pH 7.2, 5% glycerol, 1 mM PMSF, and protease inhibitor cocktail) with an equal volume of glass beads (425–600 μm; Sigma-Aldrich, St. Louis, MO, USA). After vigorously vortexing 4 times for 1 min each at 2-min intervals on ice, the lysates were centrifuged at 13,000 × *g* for 20 min at 4°C, and the supernatants were used as protein extracts. Protein concentration was determined by using Protein Dye Reagent (Bio-Rad, Hercules, CA, USA).

### MDHAR activity assay

MDHAR activity was assayed spectrophotometrically. The assay was performed at 25°C with a reaction mixture containing 50 mM potassium phosphate, pH 7.2, 0.2 mM NADH, 2 mM AsA, 1 unit of AsA oxidase (Sigma-Aldrich), and crude extract. MDHAR activity was measured by monitoring the decrease in absorbance at 340 nm resulting from NADH oxidation. Activity was calculated using an absorbance coefficient of 6.2 mM^−1^cm^−1^. One unit is the amount of enzyme that oxidizes 1 nmol of NADH per min at 25°C [26].

### Western blot analysis

To examine the expression changes in antioxidant proteins and metabolic enzymes, initial cell concentrations with 1 × 10^6^ cells per mL were inoculated into YPD medium and cultured for 6 h at 28°C until the cells reached mid-log phase and were then exposed to 20 mM H_2_O_2_ for 1 h at 28°C with shaking. The cells were harvested and lysed with glass beads as described above. Total protein (20 μg) was resolved by SDS-PAGE and electrophoretically transferred to a PVDF membrane. The membrane was blocked with 5% non-fat skim milk in Tris/HCl-buffered saline containing 0.05% Tween 20 (TBST), and then incubated at 4°C overnight with each primary antibody appropriately diluted in TBST. The blot was washed four times for 40 min with TBST and then incubated for 90 min at room temperature with appropriately diluted secondary antibody. After washing four times for 40 min with TBST, the bands were visualized using ECL Western Blotting Detection Reagent (GE Healthcare, Little Chalfont, UK). The primary antibodies used were against the following proteins: hexokinase (Hxk), glyceraldehyde-3-phosphate dehydrogenase (GAPDH), aldehyde dehydrogenase (Ald), alcohol dehydrogenase (Adh), Cu/Zn superoxide dismutase (Sod1), GR, glutathione peroxidase (Gpx), porin [27], thioredoxin isoform 2 (Trx2), Trx3, Trr, heat shock protein 104 (Hsp104), Hsp82, Hsp70 family (Ssa1 and Ssb2), Hsp60, Hsp42, Hsp30, and Hsp26. The primary and secondary antibodies were used as previously described [28]. Anti-tubulin antibody (Tub; Santa Cruz Biotechnology, Dallas, TX, USA) was used as a loading control. The anti-MDHAR antibody was produced in rabbits inoculated with purified MDHAR protein [29].

### Measurement of AsA-like content

Yeast cells were treated with or without 20 mM H_2_O_2_ for 1 h at 28°C, harvested by centrifugation, and washed twice with cold PBS. AsA-like content was determined spectrophotometrically. Yeast cells were disrupted with glass beads and 5% *m*-phosphoric acid. The homogenate was centrifuged at 12,000 × *g* for 20 min. Total AsA content was determined in a reaction mixture containing 100 μL of supernatant, 500 μL of 150 mM KH_2_PO_4_ buffer (pH 7.4) containing 5 mM EDTA, and 100 μL of 10 mM dithiothreitol (DTT) to reduce dehydroascorbate [30] to AsA. The reaction mixtures were incubated for 10 min at room temperature. Then, 100 μL of 0.5% N-ethylmaleimide (NEM) was added to remove excess DTT. Ascorbic acid was assayed in a similar manner, except that 200 μL of deionized H_2_O was substituted for DTT and NEM. In both reactions, color was developed by adding 400 μL of 10% trichloroacetic acid, 400 μL of 44% *o*-phosphoric acid, 400 μL of α, α′-dipyridyl in 70% ethanol, and 200 μL of 30 g·L^-1^ FeCl_3_. The reaction mixtures were incubated at 40°C for 1 h and quantified spectrophotometrically at 525 nm. The concentration of DHA was estimated from the difference in the concentrations of total and reduced AsA. The concentrations of AsA and DHA were quantified by generating of a standard curve using AsA from 1 to 10 μM mL^-1^ [31].

### Cellular redox state assay

Cells were cultured for 6 h at 28°C until they reached mid-log phase and then exposed to 20 mM H_2_O_2_ for 1 h at 28°C. The cells were harvested and lysed with glass beads. The intracellular hydroperoxide level was determined by ferrous ion oxidation in the presence of the ferric ion indicator xylenol orange [32]. Fifty microliters of crude cell extract were added to 950 μL of FOX reagent (100 μM xylenol orange [water-soluble form; Sigma-Aldrich], 250 μM ammonium ferrous sulfate, 100 mM sorbitol, and 25 mM sulfuric acid). The mixture was incubated at room temperature for 30 min and then centrifuged to remove any flocculated material before measuring the absorbance at 560 nm. Hydroperoxide concentration is reported as μg per mg of protein. For the fluorescence assay, yeast cells were treated with 0.1 mM dichlorodihydrofluorescein diacetate (DCFHDA) and 0.1 mM dihydrorhodamine 123 (DHR 123) for 20 min at 28°C with shaking before treatment with 10 mM or 20 mM H_2_O_2_ for 1 h at 28°C. Treated cells were washed twice with PBS and visualized by fluorescence microscopy (excitation, 488 nm; emission, 525 nm) [33]. Protein oxidation was determined using the Protein Carbonyl Content Assay Kit (Sigma-Aldrich) according to the manufacturer’s protocol.

### Laboratory glucose-based batch fermentation

Cells (initial concentration adjusted to 1 × 10^6^ cells per mL) grown at 30°C overnight were inoculated into YG medium containing 20% glucose and 1% yeast extract and then fermented under aerobic conditions on a shaker (160 rpm) for 72 h at 30°C. The alcohol concentration was determined based on the percentage (v/v) of alcohol in the distillate after fermentation, which was measured using an alcohol hydrometer (REF 503; Korins, Seoul, Korea). The residual glucose concentration was measured with a hand-held Refractometer N1 (Atago, Tokyo, Japan). Alcohol and residual glucose were measured after removing cell debris by centrifugation (2,000 × *g*, 3 min). Growth was monitored for 24 h under the same conditions. To investigate cell survival during fermentation, cells were harvested at three time points (24, 48, and 72 h) and serially diluted to 10^−9^ with deionized distilled water, and then 5 μL of each dilution was plated onto YPD agar, incubated for 3 days at 30°C, and then photographed. During fermentation, MDHAR expression was analyzed at various time points by western blotting as described above.

### Statistical analysis

Significant differences in the measured parameters were identified using Origin Pro 8.0. Means were considered to be significantly different when *P* < 0.05. All experiments were independently performed at least three times, and the results are expressed as the mean ± standard deviation (SD). The results of the spotting, growth kinetics, and fermentation assays are representative of at least two independent experiments performed under identical conditions.

## Results

### Generation of *OsMDHAR*-expressing recombinant yeast and their stress response to an oxidant

**Fig 1:**
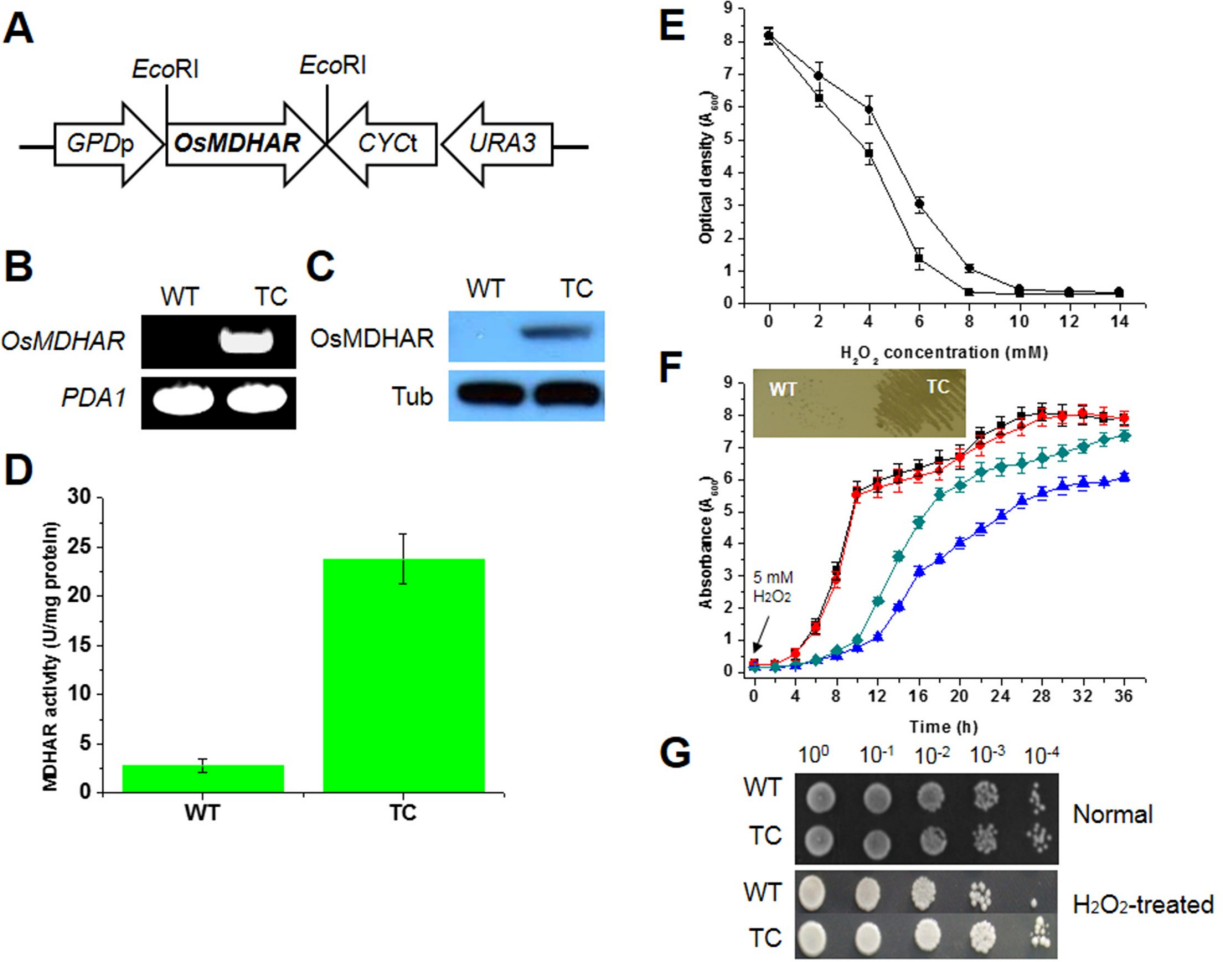
Construction of an *OsMDHAR-*expressing yeast vector and the stress response of *OsMDHAR-*expressing yeast to hydrogen peroxide. (A) Schematic diagram of the p426GPD::OsMDHAR construct. The *OsMDHAR* gene (approximately 1.5 kbp) was subcloned to generate the p426GPD::OsMDHAR construct with *OsMDHAR* under the control of the constitutive *GPD* promoter. Semi-quantitative RT-PCR (B), immunoblotting (C), and an enzymatic assay (D) were performed to examine whether *OsMDHAR* is expressed in this yeast strain. *PDA1* and tubulin (Tub) were used as housekeeping controls for RT-PCR and western blotting, respectively. The molecular size of the PCR product and molecular weight of the detected band were approximately 494 bp and 47 kDa, respectively. Stress tolerance to hydrogen peroxide was evaluated by cell survival, growth kinetics, and spotting assays. (E) To monitor cell viability, yeast cells precultured in YPD medium were inoculated into fresh YPD medium and exposed to different concentrations of H_2_O_2_ for 16 h at 28°C. Then, the optical density at 600 nm (OD_600_) was measured. Circles, cells transformed with p426GPD-*OsMDHAR* (TC cells); squares, wild-type (WT) cells transformed with an empty vector. (F) For the growth kinetics assay, precultured yeast cells were inoculated into YPD medium containing 5 mM H_2_O_2_, and the OD_600_ was measured at 2-h intervals for 36 h. A streaking assay was also performed, in which mid-log phase yeast cells (OD_600_ ≈ 2.0) were streaked onto YPD agar plates supplemented with 5 mM H_2_O_2_. WT (squares) and TC (circles) cells in the absence of 5 mM H_2_O_2_; WT (upward triangles) and TC (diamonds) cells in the presence of 5 mM H_2_O_2_. (G) Mid-log phase yeast cells were exposed to 20 mM H_2_O_2_ for 1 h with shaking, and serially diluted with YPD medium. A 5-μL aliquot of each dilution was spotted onto YPD agar plates.

A cDNA containing the open reading frame (ORF) of *MDHAR* from *O*. *sativa* (*OsMDHAR*) was subcloned into the yeast expression vector p426GPD, which allows constitutive expression of a target gene under the control of the yeast *GPD* (glyceraldehyde-3-phosphate dehydrogenase) promoter (Fig 1A). To determine whether the *OsMDHAR* gene is expressed in yeast, semi-quantitative RT-PCR and immunoblotting were performed. A single DNA fragment of approximately 494 bp corresponding to a region within *OsMDHAR* was amplified from cells transformed with the p426GPD-*OsMDHAR* construct (TC), whereas no amplification signal was detected in wild-type cells transformed with vector alone (Fig 1B). To investigate whether the OsMDHAR protein is expressed, western blotting and enzymatic activity assays were performed. As shown in Fig 1C, immunoblotting analysis using an antibody raised against MDHAR showed a single band at 47 kDa derived from the *MDHAR* transgene in the crude extract prepared from the TC cells, but not in extract prepared from WT cells. The enzymatic activity of MDHAR in the TC cells was approximately 10-fold higher than that in the WT cells (Fig 1D). There was a clear correlation between the *OsMDHAR* mRNA level and the MDHAR protein level. To determine if *OsMDHAR* is related to stress tolerance in yeast, a cell survival assay was used. In this assay, the optical density of the TC cells was higher than that of the WT cells at a variety of H_2_O_2_ concentrations. The WT cells were completely inhibited in 8 mM H_2_O_2_, whereas the TC cells were inhibited in 10 mM H_2_O_2_ (Fig 1E). To confirm these results, growth kinetics, streaking, and spotting assays were performed. In the growth kinetics assay, the TC cells recovered more rapidly than the WT cells did in the presence of 5 mM H_2_O_2_ for the indicated times. However, under normal conditions, there was no difference between the cells (Fig 1F). The streaking assay results supported the growth rate findings (Fig 1F, boxed panel). The spotting assay also demonstrated that the TC cells had acquired increased stress tolerance when they were exposed to 20 mM H_2_O_2_ for 1 h at 28°C with shaking, compared to that of the WT cells (Fig 1G). These results suggest that *OsMDHAR* expressed in *S*. *cerevisiae* as a eukaryotic model system effectively enhances stress tolerance when the cells are challenged with oxidative stress.

### Expression of cell rescue proteins and the redox state under oxidative stress

**Fig 2:**
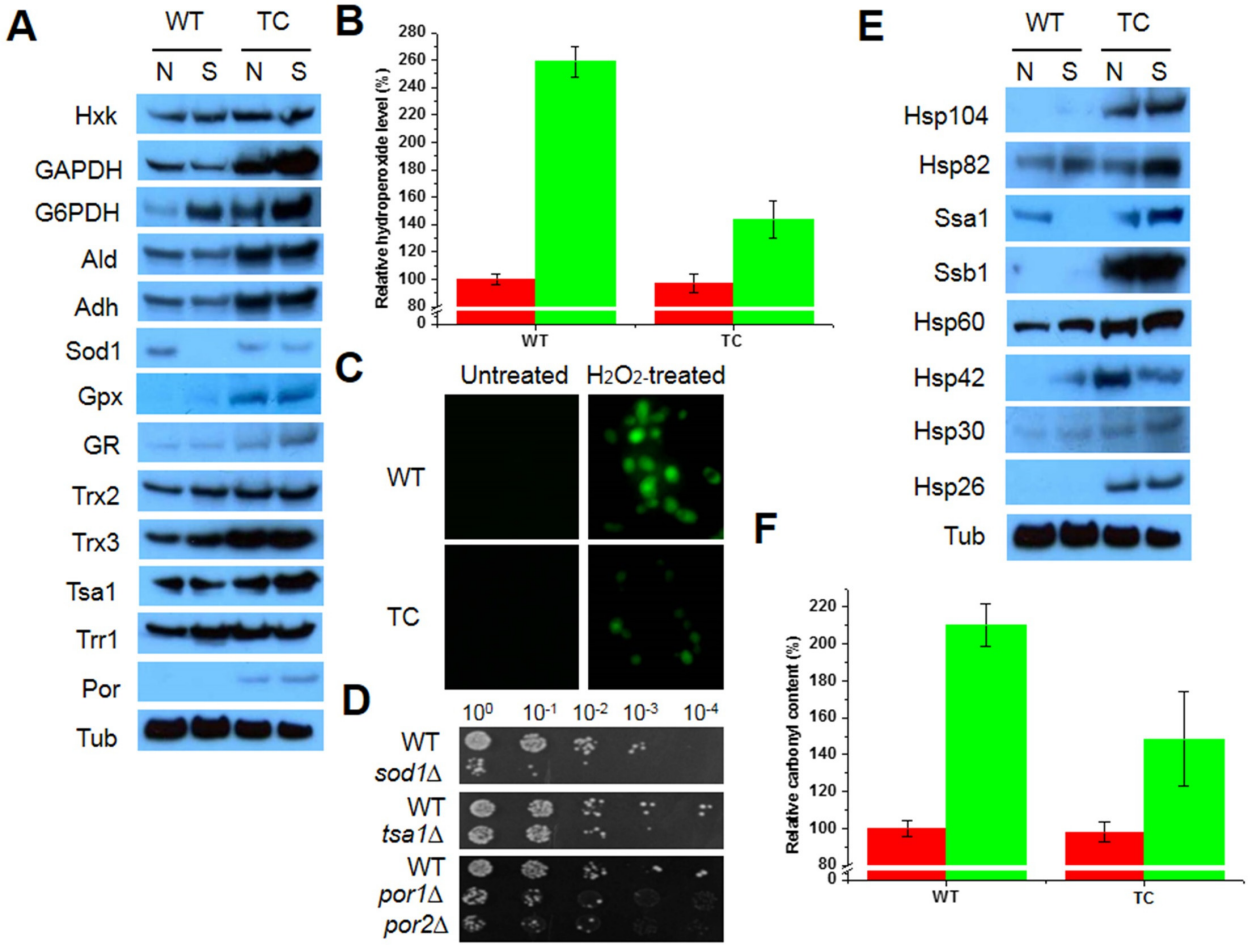
Analyses of cell rescue proteins, redox state, and protein oxidation under oxidative conditions. (A) Expression changes in antioxidant and metabolic enzymes in mid-log phase yeast cells exposed to 20 mM H_2_O_2_ for 1 h with shaking. Tubulin (Tub) was used as a loading control. (B) Hydroperoxide levels in TC cells in the absence (red bar) and presence (green bar) of 20 mM H_2_O_2_ were assessed using FOX reagent and were calculated relative to that in WT cells grown under normal conditions, which was set to 100%. (C) Mid-log phase yeast were exposed to 20 mM H_2_O_2_ for 1 h at 28°C with shaking. Redox state was analyzed by measuring DCFHDA oxidation as an indicator of cytosolic ROS. (D) Sensitivity of mutants (*sod1Δ*, *tsa1Δ*, *por1Δ*, and *por2Δ*) to oxidative stress. Yeast cells (OD_600_ ≈ 1.0) were exposed to 10 mM H_2_O_2_ for 1 h at 28°C with shaking, serially diluted with YPD medium, spotted onto YPD agar plates, and incubated for 2–3 days. (E) Expression changes in molecular chaperones in mid-log phase yeast cells exposed to 20 mM H_2_O_2_ for 1 h with shaking. Tubulin (Tub) was used as a loading control. (F) Protein carbonylation in yeast cells exposed to 20 mM H_2_O_2_ for 1 h was calculated relative to that in WT cells under normal conditions, which was set to 100%. Red bar, normal conditions; green bar, H_2_O_2_ treatment; WT, yeast cells with an empty vector; TC, *OsMDHAR*-expressing yeast cells; N, normal conditions; S, H_2_O_2_ treatment.

Compared to WT cells, TC cells showed increased survival following acquired tolerance under oxidative stress. To further understand the mechanism underlying the improved tolerance of TC cells, expression changes in rescue proteins were assessed by western blotting after exposure to oxidative stress. A wide range of proteins was upregulated in TC cells during oxidant challenge compared to the expression levels in WT cells (Fig 2). The expression of several metabolic enzymes, including Hxk, GAPDH, G6PDH, Ald, and Adh, was increased in the TC cells under oxidative stress, compared to that in WT cells. In addition, numerous antioxidant enzymes were upregulated, including Sod1, Gpx, GR, Trx2, Trx3, Tsa1, and Por, in TC cells. However, cytosolic Trr1 expression was similar in TC and WT cells grown under oxidative stress (Fig 2A). High expression of metabolic enzymes and antioxidant enzymes resulted in improved redox homeostasis in TC cells. The hydroperoxide level in TC cells was approximately 1.8-fold higher than that in WT cells cultured in the presence of H_2_O_2_; however, there was no difference between TC and WT cells cultured under normal conditions (Fig 2B). These results were supported by DCFHDA fluorescence, an indicator of cytosolic hydroperoxide, as DCFHDA fluorescence was lower in TC cells than in WT cells, although there was a change in signal intensity in both cells under oxidative stress (Fig 2C). Expression of Sod1, Tsa1, Por1, and Por2 was inhibited or unchanged in WT cells under oxidative stress, compared to the expression levels in TC cells. Mutants with deletions in antioxidant enzymes (*sod1Δ*, *tsa1Δ*, *por1Δ*, *por2Δ*, and *ara2Δ*) were hypersensitive to oxidative stress (Fig 2D). Unexpectedly, the recovery of *por1Δ* cells in growth kinetics and spotting assays was slower than that of WT cells without an empty vector (BY cells) in the presence of H_2_O_2_; however, there was no difference between these cells under normal condition (S1A and S1B Fig). *Por1Δ* cells displayed increased cytosolic and mitochondrial hydroperoxide levels under stress compared to the levels in BY cells (S1C Fig). In addition, *por1Δ* cells were sensitive to a wide range of stressors, including oxidants (menadione [MD] and *tert*-butylhydroperoxide [*t*-BOOH]), heat shock, metals (copper, iron, and zinc), heavy metals (aluminum and cobalt), acids (sulfuric acid and salicylic acid), and high salinity (NaCl) (S1D Fig). The expression of various molecular chaperones, HSPs, and cofactors was increased in TC cells under oxidative stress. The highly expressed proteins included Hsp104, Hsp82, Hsp60, Hsp30, Hsp26, and two members of the Hsp70 family (Ssa1 and Ssb1). Although the expression of Hsp42 in TC cells was lower under oxidative stress than under normal conditions, it was still higher in TC cells than in WT cells (Fig 2E). This high expression of chaperone proteins in TC cells resulted in decreased protein oxidation, and the protein carbonyl content of TC cells was lower than that of WT cells (Fig 2F). Thus, TC cells express a range of rescue proteins that enable them to respond to oxidative stress by improving redox homeostasis and proteostasis following the decreases in cellular hydroperoxide and protein oxidation levels.

### AsA-like content and its effect on H_2_O_2_

**Fig 3:**
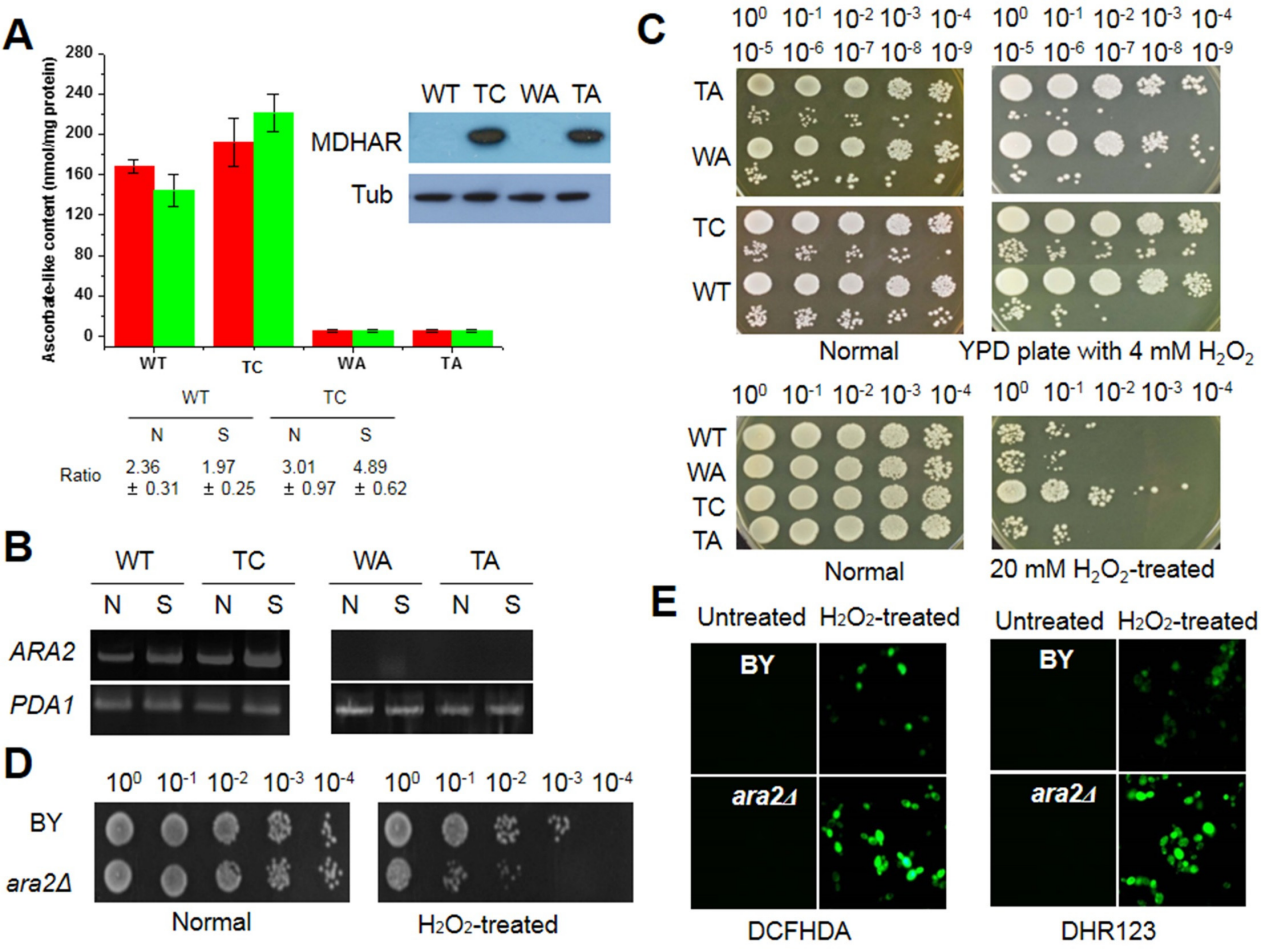
Stress response related to ascorbate (AsA)-like molecules. (A) AsA-like content in yeast cells exposed to 20 mM H_2_O_2_ for 1 h was analyzed and is shown as nmol per mg protein. The ratio shown is that of the reduced form to oxidized form. (B) *ARA2* expression was evaluated by semi-quantitative RT-PCR. *PDA1* was used as a control. (C) Oxidative stress response of yeast cells in the absence and presence of *ARA2*. Mid-log phase cells were serially diluted, and 5 μL of the diluted solutions were spotted onto YPD agar plates containing 4 mM H_2_O_2_ (upper panels). Mid-log phase cells were treated with 20 mM H_2_O_2_ for 1 h with shaking, diluted with YPD medium, and spotted onto YPD agar plates. The plates were incubated for 2–3 days and photographed. (D) Stress sensitivity of *ara2Δ* yeast cells, in which the erythroascorbate (EAA) biosynthesis gene was deleted. Yeast cells (A_600_ ≈ 1.0) were exposed to 10 mM H_2_O_2_ for 1 h at 28°C with shaking, serially diluted with YPD medium, spotted onto YPD agar plates, and incubated for 2–3 days. (E) The redox state of *ara2Δ* yeast cells under oxidative conditions. Yeast cells (OD_600_ ≈ 1.0) were exposed to 10 mM H_2_O_2_ for 1 h after DCFHDA and DHAR 123 treatment for 30 min and washed twice with phosphate-buffered saline (PBS). Probe intensity was observed by fluorescence microscopy. BY, wild-type cells without an empty vector; *ara2Δ*, cells with a deletion of the EAA biosynthetic gene *ARA2*; WT, wild-type yeast cells with an empty vector; TC, yeast cells with p426GPD::OsMDHAR; WA, *ara2Δ* yeast cells with an empty vector; TA, *ara2Δ* yeast cells with p426GPD::OsMDHAR; N, normal conditions; S, in the presence of H_2_O_2_.

AsA and its analogue erythroascorbate (EAA), are antioxidant molecules that act as scavengers of free radicals and are essential for resistance and adaptation to abiotic stresses in yeast [34]. AsA is the most abundant antioxidant molecule in the cell, thus it is a good target for investigation of the intrinsic ability of *OsMDHAR*-expressing cells to eliminate free radicals. Since AsA-like content is an indication of the capacity of cells to manage exogenous stressors, we investigated a yeast strain in which the EAA biosynthesis-related gene *ARA2* was deleted (*ara2Δ*). Deletion of *ARA2* was confirmed by evaluating empty vector (p426GPD)- and OsMDHAR::p426GPD-transformed *ara2Δ* cells (WA and TA, respectively) by immunoblotting (Fig 3A, boxed panel). The EAA content of exponential phase WT, WA, TA, and TC cells was analyzed in YPD-grown cultures in the absence and presence of 20 mM H_2_O_2_. As shown in Fig 3A, significant differences in EAA content were observed between WT and TC cells. Although the EAA levels before and after stress were unchanged, the EAA pool in TC cells was approximately 1.3-fold higher than that in WT cells. In contrast, EAA was not detected in WA or TA cells. The accumulation of EAA in TC cells corresponded to the ratio of the reduced form to oxidized form in the presence of H_2_O_2_. To examine whether *ARA2* is required for the increased EAA pool, *ARA2* expression was measured by semi-quantitative RT-PCR. *ARA2* expression was highest in TC cells exposed to oxidative stress, whereas it increased in WT cells under the same condition. In contrast, no expression was detected in WA or TA cells (Fig 3B). The importance of EAA in the response to oxidative stress was examined by a spotting assay and redox state analysis. Recovery capacity was higher in TC cells than in the other tested cells (WT, WA, and TA) in the presence of H_2_O_2_; however, no difference in growth kinetics was observed among the four cell types under normal conditions (Fig 3C). *Ara2Δ* cells were hypersensitive to oxidative stress compared to the sensitivity of BY cells (Fig 3D), and they displayed increased hydroperoxide levels in the cytosol and mitochondria, as measured using DCFHDA and DHR124 as indicator probes, respectively (Fig 3E). These results imply that EAA plays an important role in the oxidative stress response, and that in WT cells, the levels of EAA are insufficient to promote stress tolerance, compared to the levels in TC cells. Based on these results, in subsequent experiments, we tested the effects of exogenous AsA or its analogue IAA on the stress response of yeast cells. Although WT cells were more sensitive to oxidative stress than TC cells, spotting assays showed that WT cells reached the same growth levels as TC cells in the presence of exogenous AsA or IAA, which is indicative of adaptation capacity under oxidative stress (S2A Fig, upper panel). Supplementation with these molecules led to enhanced stress tolerance in *ara2Δ* cells under oxidative stress (S2A Fig, lower panel). *Ara2Δ* cells were also sensitive to various stressors, including heat shock, MD, *t*-BOOH, ethanol, NaCl, cadmium, zinc, and lactic acid (S2B Fig).

### Effect of *OsMDHAR* expression under batch fermentation

**Fig 4:**
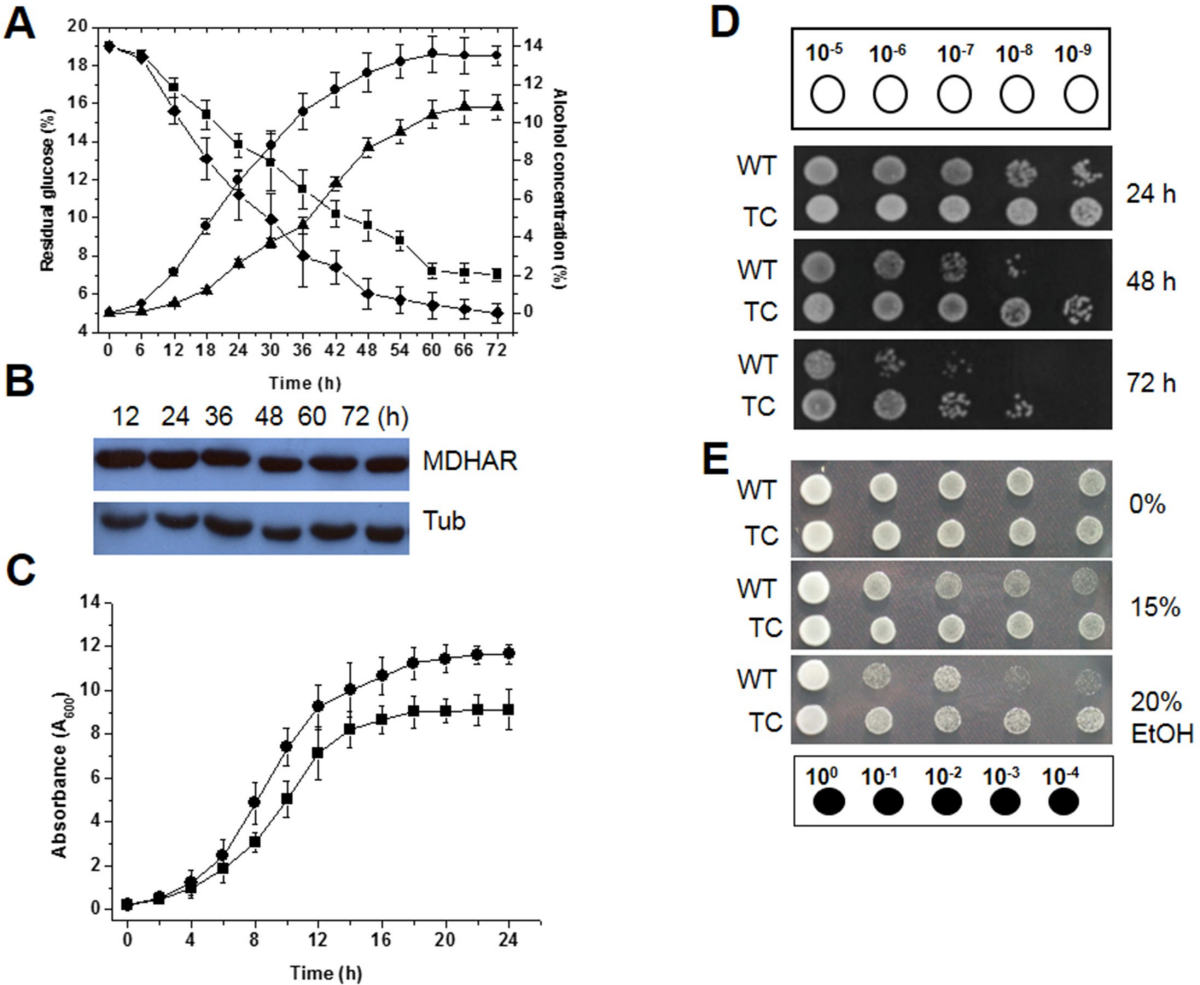
Fermentative capacity and the survival of *OsMDHAR*-expressing yeast cells during batch fermentation. (A) Fermentative capacity was analyzed by measuring the alcohol (AC) and residual glucose (RG) concentrations in YG medium after fermentation for 72 h at 30°C. Upward triangles, AC of WT cells; circles, AC of TC cells; squares, RG of WT cells; diamonds, RG of TC cells. (B) Time-dependent *OsMDHAR* expression during batch fermentation was evaluated by western blotting. Tubulin (Tub) was used as a loading control. (C) Growth kinetics during fermentation was assessed by measuring the OD_600_ at 2-h intervals for the indicated time. Squares, WT cells; circles, TC cells. (D) Cell viability during fermentation at 30°C was assessed by a spotting assay. Cells were harvested after 24 h (upper panel), 48 h (middle panel), and 72 h (lower panel) of fermentation and serially diluted to 10^−9^. A 5-μL aliquot of each diluted solution was spotted onto YPD agar plates. After incubation for 3 days, the plates were photographed. (E) Stress response to ethanol. Mid-log phase yeast cells (OD_600_ ≈ 2.0) were exposed to different concentrations of ethanol (0, 15%, and 20%) for 1 h, serially diluted, and spotted onto YPD agar plates. WT, yeast cells with an empty vector; TC, *OsMDHAR*-expressing yeast cells.

Since TC cells expressing *OsMDHAR* were more tolerant to oxidative stress than WT cells, these cells were tested in glucose-based batch fermentation in YG medium under aerobic conditions. Distinct differences in alcohol yield and residual glucose content were observed between TC and WT cells at 30°C, which is the temperature that is typically used for industrial fermentation. During fermentation for 72 h at 30°C, the alcohol yield of TC cells was approximately 25% higher than that of WT cells. The final alcohol concentration was approximately 13.5% and 10.2% with TC cells and WT cells, respectively, and the residual glucose concentration was inversely proportional to the alcohol concentration during fermentation (Fig 4A). *OsMDHAR* expression in the TC cells during fermentation was confirmed by western blotting, although protein expression decreased slightly over time (Fig 4B). In addition, distinct differences in growth kinetics and cell survival were observed between TC and WT cells during fermentation. The growth rate in early fermentation until stationary phase was higher in TC cells than in WT cells (Fig 4C). In particular, the survival of TC cells was time-dependently higher than that of WT cells during fermentation in YG medium for 72 h, although cell viability decreased gradually with time (Fig 4D). Next, to examine the response to various concentrations of ethanol, yeast cells were grown to log phase (OD_600_ = 2.0), and then exposed to 15% or 20% ethanol for 1 h with shaking. After exposure, the cultures were serially diluted and spotted onto YPD agar plates. TC cells were more tolerant to ethanol than WT cells (Fig 4E). In addition, TC cells expressing *OsMDHAR* were more resistant to ROS-induced oxidative stress (MD, *t*-BOOH, copper, iron, cadmium, and sodium dodecyl sulfate [SDS]) than WT cells (S3 Fig). Therefore, our findings indicate that *OsMDHAR* expression enhances fermentative capacity at moderate temperatures, which is an important factor for increased alcohol yield during fermentation.

## Discussion

We evaluated an *OsMDHAR*-expressing yeast strain and compared it a control WT strain to determine if *OsMDHAR* could improve the stress tolerance of *S*. *cerevisiae* as a eukaryotic model system. The viability of cells transformed with p426GPD::OsMDHAR (TC cells), which constitutively express *OsMDHAR* under the control of the *GPD* promoter, was inhibited by a higher concentration of H_2_O_2_, than the concentration that inhibited the viability of WT cells with an empty vector (Fig 1). In growth kinetics, streaking, and spotting assays, *OsMDHAR*-expressing TC cells were more resistant to H_2_O_2_ than WT cells. We measured the stress tolerance of TC cells containing recombinant *OsMDHAR* in the presence of 5 mM or 20 mM H_2_O_2_. *OsMDHAR* expression in yeast enhanced the acquired resistance to H_2_O_2_ (Fig 1) and to a wide range of ROS-induced oxidative stressors, including oxidants (MD and *t*-BOOH), redox-active metals (iron, copper, and cobalt), a non-redox-active metal (cadmium), SDS, and ethanol (S3 Fig). Although no previous studies have established a relationship between *OsMDHAR* expression and the stress response in yeast, the overexpression of eukaryotic antioxidant genes has been shown to improve stress resistance in both prokaryotes and eukaryotes [35–37].

To investigate whether the improved tolerance to oxidative stress in TC cells was due to an *OsMDHAR*-mediated response, the expression of various rescue proteins was analyzed. Under oxidative stress, TC cells upregulated several metabolic enzymes, including Hxk, GAPDH, Adh, Ald, and G6PDH, and antioxidant enzymes, including Sod1, Gpx, GR, Trx2, Trx3, Tsa1, and Por (Fig 2A). In contrast, Trr1 expression was the same in TC and WT cells under stress. The expression of most proteins was stress-dependently induced in TC cells. The major stress-protection mechanisms include synthesis of antioxidant proteins, proteins involved in metabolic and pentose phosphate pathways and molecular chaperones, and the accumulation of compatible solutes and hydrophylins as well as permeability adaptation of the plasma membrane [18]. Upregulation or overexpression of stress-related genes (*SOD*, *CAT*, *GPX*, *TSA1*, and *TRX*) enhances acquired resistance to ROS-induced oxidative stresses, including oxidants (O_2_*^−^, H_2_O_2_, and MD), ethanol, and lactic acid, by improving redox homeostasis following the reduction of oxidative damage through cross-protection between target proteins and other antioxidant enzymes in *S*. *cerevisiae* [38–42], whereas the direct interactions between H_2_O_2_ and other antioxidant proteins play a secondary role by protecting ribosomal proteins from stress-induced aggregation through the function of CAT and other peroxiredoxins [43,44]. In addition, the TRX system (TRX plus TRR) can act as an alternative system to reduce GSH disulfide (GSSG) in the presence of NADPH in *glr1*-deficient yeast cells and buffer oxidative stress [45]. Tomato *MDHAR*- and *Arabidopsis thaliana* MDHAR (*AtMDHAR1*)-overexpressing transgenic plants showed acquired tolerance against abiotic stressors (chilling and high temperature stresses, ozone, salt, polyethylene glycol, and radiation) by improving redox homeostasis through lowering the levels of hydrogen peroxide and lipid peroxidation and increasing the net photosynthetic rate, PSII effective quantum yield (*Fv/Fm*), fresh weight, and antioxidant’s enzyme activities (DHAR, GR, SOD and peroxidases) by elevating the AsA level compared to in WT plants [11,12,26]. In contrast, downregulation or repression of these antioxidant systems leads to a number of stress-dependent phenotypes, including genome instability [46], slow growth, and increased sensitivity to ROS-generating agents such as H_2_O_2_, which accelerates aging, auxotrophy, and cell death [47]. In addition, the suppression of proteins involved in the pentose phosphate pathway (i.e., G6PDH) and another pathway (isocitrate dehydrogenase) important for reducing power production increased the sensitivity to oxidative stress following an imbalance in NADPH homeostasis [47–49]. Furthermore, TC cells showed increased porin (Por) expression under oxidative stress (Fig 2A). Mitochondrial function affects redox homeostasis, which is mediated primarily by the voltage-dependent anion channel (VDAC; porin pore). VDAC releases superoxide anions from the mitochondria into the cytosol and increases cytosolic ROS [50]. However, *por1Δ* cells showed increased cytosolic and mitochondrial ROS levels (S1 Fig). Taken together, these findings highlight the importance of *OsMDHAR*-mediated mechanisms in maintaining ROS at non-toxic levels and cross-protection through the activation of various antioxidant enzymes.

In addition to metabolic and antioxidant enzymes, we observed increased expression of various HSPs, molecular chaperones, and cofactors, including Hsp104, Hsp90 (Hsp82), the Hsp70 family members (Ssa1 and Ssb1), Hsp60, Hsp42, Hsp30, and Hsp26, in TC cells under oxidative stress. (Fig 2E). Accumulation of chaperone proteins resulted in decreased stress-induced injury (Fig 2F). ROS-induced oxidative stress causes aggregation and inactivation of nascent proteins due to misfolding, which at high levels, can lead to neurodegenerative disease, cancer, viral infection, and cell death. To protect themselves from oxidative damage, yeast cells have evolved a wide range of molecular chaperones and cofactors, including Hsps that play a central role in protein homeostasis [51,52]. In eukaryotes, the cooperative functions of Hsp104, Ssa1, Hsp42, and Hsp26 rescue aggregated or misfolded proteins produced by oxidative stress through several folding process, such as general folding, nascent chain folding, and post-import folding following conformational changes associated with Fe/S proteins and substrate binding [51–54]. Hsp90 reactivates unfolded or misfolded proteins (referred to as client proteins) by interacting or assembling with co-chaperones and cofactors [51,55], which also participate in different important cellular functions, such as growth and differentiation, as well as stress responses [56]. As described above, sHsps such as Hsp42 and Hsp26 play a critical role in protein solubility when ROS-induced oxidative stress leads to general cytosolic protein unfolding; however, other sHsps, including Hsp30, have not been well characterized even though they also appear to play a role in stress tolerance [52]. Based on these observations, our results indicate that *OsMDHAR* expression leads to a marked accumulation of chaperone proteins, which facilitates protein refolding and minimizes oxidative damage by maintaining the balance of chaperone machinery systems, thus improving proteostasis under oxidative stress. However, the relationship between *OsMDHAR* and Hsp expression remains unclear.

*OsMDHAR* expression in TC cells increased the ratio of reduced and oxidized forms following the accumulation of the AsA-like molecule EAA under oxidative stress (Fig 3A). TC cells were more tolerant to ROS-induced oxidative stress than WT cells (Fig 3C). These results show that EAA in WT cells is insufficient to induce the necessary machinery for stress tolerance compared to TC cells. Treatment of WT cells with AsA or its analogue IAA improved the stress response, such that it was similar to that of TC cells. Supplementation with AsA or IAA also enhanced acquired tolerance in WA cells, although the stress resistance of WA cells was lower than that of TC and WT cells (S2 Fig). These results show that EAA as an AsA analogue plays an important role in tolerance to oxidative stress. From this perspective, the increase in the AsA-like pool is mainly the result of EAA recycling through OsMDHAR as well as EAA biosynthesis following upregulation of *ARA2* under unfavorable conditions. MDHAR, which is widely found in eukaryotes, is an important antioxidant that functions as a key enzyme for maintaining AsA pools. AsA is synthesized by all higher plants and nearly all higher animals, with the exception of some species, including humans, primates, guinea pigs, some birds, and fish [57]. AsA and its analogue EAA are the most abundant water-soluble free radical scavenger antioxidants in living organisms. AsA serves as a cofactor for enzymes involved in hormone biosynthesis and is involved in the recycling of antioxidants such as AsA, α-tocopherol, and phenolics [49]. The precise mechanisms underlying these functions are not yet known. In *Candida albicans*, EAA peroxidase is capable of detoxifying H_2_O_2_ by converting it to water through oxidation of EAA, which increased tolerance to oxidative stress [34]. After participating in the reaction, EAA can be recycled by several different mechanisms. The EAA radical, produced following EAA oxidation, can be recycled following reduction by OsMDHAR or mitochondrial NADH-dependent cytochrome b_5_ reductase [58]. In bioassays using tobacco hornworm (*Manduca sexta*), EAA induced larval growth almost as well as AsA because AsA can be converted to EAA by a ubiquitin-mediated process [59]. Because of this OsMDHAR-mediated recycling, EAA content and its redox state are maintained, which is critical under ROS-induced oxidative stress. Additionally, EAA plays a role in cell growth and development, as well as in response to oxidative stress.

To further elucidate the functions of *OsMDHAR*, the glucose-based batch fermentative capacity of TC and WT cells was compared at 30°C (a typical industrial temperature) because Brazil, the United States, and Korea produced approximately 21.6, 50.3, and 0.15 billion gallons of bioethanol, respectively in 2012, which is more than double that produced in 2007 [60]. During batch fermentation at 30°C, *OsMDHAR* was constitutively expressed until the end of fermentation (Fig 2B). The alcohol concentrations produced by fermentation using TC and WT cells were 13.5% (0.71 ± 0.02 g·g^−1^) and 10.2% (0.53 ± 0.03 g·g^−1^), respectively (Fig 4A), and the alcohol concentration in TC cells was approximately 25% (0.18 g·g^−1^) higher than that in WT cells. In general, the theoretical ethanol yield from glucose fermentation by *S*. *cerevisiae* is 10–11% (0.50–0.55 g·g^−1^) [61]. Interestingly, the alcohol yield of TC cells in this study was 9.4% (0.24 g·g^−1^) higher than of WT cells reported previously [61]. Survival during fermentation at 30°C was also higher for TC cells than for WT cells (Fig 4D). The alcohol concentration of *OsMDHAR*-expressing TG yeast (13.5%; 0.71 ± 0.02 g·g^−1^) was higher than that of *CaDHN*- (13.3%; 0.63 ± 0.03 g·g^−1^) and *OsTPX*-expressing TG yeasts (12.9%; 0.61 ± 0.01 g·g^−1^); which were under the control of the same promoter) [62,63]. Current bioethanol production relies on agricultural products, including dextrose derived from corn in the US and sucrose derived from sugar in Brazil [60]. The ethanol produced during sugar-based fermentation affects cell viability, growth rate, and fermentation capacity and quality. To overcome these problems, genetically modified yeast strains have been introduced. Compared to *S*. *cerevisiae*, other yeasts have some limitations, such as low ethanol yield and poor ethanol tolerance in typical glucose-based fermentation [64,65]. *Saccharomyces* spp. is most attractive microorganism for fermenting sugars to ethanol. Besides its innate properties, overexpression of stress-related genes could be a useful method for developing ethanol-tolerant yeasts because ethanol toxicity is highly related to ROS generation. For example, overexpression of *TRX2* increased fermentation capacity and wine yield and quality by preventing the oxidative damage to glycolytic and fermentation proteins (Ahp1) through cross-protection of transcription factor (Yap1) and antioxidant enzymes (Sod1, Sod2, and CAT) in industrial wine yeast during biomass-based batch- and fed-batch fermentation [42,66]. Upregulation of *G6PDH* (*ZWF1*) [36], *MPR1* [67], and *PAD* [68] conferred tolerance to ethanol by enhancing NADPH and redox homeostasis or by detoxifying inhibitory compounds. To obtain a high ethanol yield in fermentation, improved *S*. *cerevisiae* strains that are tolerant of ethanol and different metabolic environments are required, such as the *OsMDHAR*-expressing transgenic yeast developed herein. As shown, *OsMDHAR* expression in TC cells increased tolerance to ethanol and sugar-induced osmotic and physiochemical stresses.

In conclusion, high expression of *OsMDHAR* in a transgenic yeast strain increased acquired tolerance to ROS-induced oxidative stress, such as that generated by H_2_O_2_, by maintaining balanced cellular redox homeostasis, proteostasis, and the pool of EAA as an AsA-like molecule. It is possible that the expression of *OsMDHAR* in yeast enhanced tolerance to oxidative stress and that all antioxidant defense mechanisms act synergistically, cross-protecting the cells at different levels and functioning to generate a fast and effective defense response under oxidative stress. In addition, heterologous *OsMDHAR* expression enhanced tolerance to high concentrations of ethanol, resulting in improved fermentative capacity. The genetically modified yeast used in this study show potential for use in the fermentation industry to improve ethanol yield. Further studies are needed to elucidate the mechanism underlying increased ethanol tolerance and yield in *OsMDHAR*-expressing *S*. *cerevisiae* during fermentation.

## Supporting Information

S1 Fig Stress sensitivity and redox state of *por1*△ yeast cells under oxidative stress. (DOCX)

S2 Fig Exogenous effect of ascorbate and its analogue on the stress sensitivity of *ara2Δ* yeast cells. (DOCX)

S3 Fig Response of *OsMDHAR*-expressing yeast cells to abiotic stressors. (DOCX)

S1 Methods Cellular response and redox state in *por1Δ* yeast cells under oxidative stress. (DOCX)

S2 Methods Cellular response and redox state in *ara2*△ yeast cells under oxidative stress and exogenous effect of AsA and its analogue. (DOCX)

S3 Methods Cellular response and redox state in yeast under oxidative stress. (DOCX)

S1 Table Oligonucleotide sequence used in this study. (DOCX)

S2 Table Strains and plasmids used in this study. (DOCX)
