# Correction: Potential Application of the Oryza sativa Monodehydroascorbate Reductase Gene (OsMDHAR) to Improve the Stress Tolerance and Fermentative Capacity of Saccharomyces cerevisiae

**DOI:** 10.1371/journal.pone.0167843

**Published:** 2016-12-01

**Authors:** Il-Sup Kim, Young-Saeng Kim, Yul-Ho Kim, Ae Kyung Park, Han-Woo Kim, Jun-Hyuck Lee, Ho-Sung Yoon

The fourth and sixth authors’ names are spelled incorrectly. The correct names are Ae Kyung Park and Jun-Hyuck Lee respectively. The correct citation is: Kim I-S, Kim Y-S, Kim Y-H, Park A.K, Kim H-W, Lee J.H, et al. (2016) Potential Application of the *Oryza sativa* Monodehydroascorbate Reductase Gene (*OsMDHAR*) to Improve the Stress Tolerance and Fermentative Capacity of *Saccharomyces cerevisiae*. PLoS ONE 11(7): e0158841. doi:10.1371/journal.pone.0158841. The correct contributions are: Performed the experiments: I-SK Y-SK AKP H-WK JHL.
