# Supplementary material for: Potential Application of the Oryza sativa Monodehydroascorbate Reductase Gene (OsMDHAR) to Improve the Stress Tolerance and Fermentative Capacity of Saccharomyces cerevisiae
Source: PLoS One. 2016 Jul 8;11(7):e0158841. doi: 10.1371/journal.pone.0158841 (PMC4938589; doi:10.1371/journal.pone.0158841)
Supplement: S1 Fig — (DOCX) [file pone.0158841.s001.docx]

**
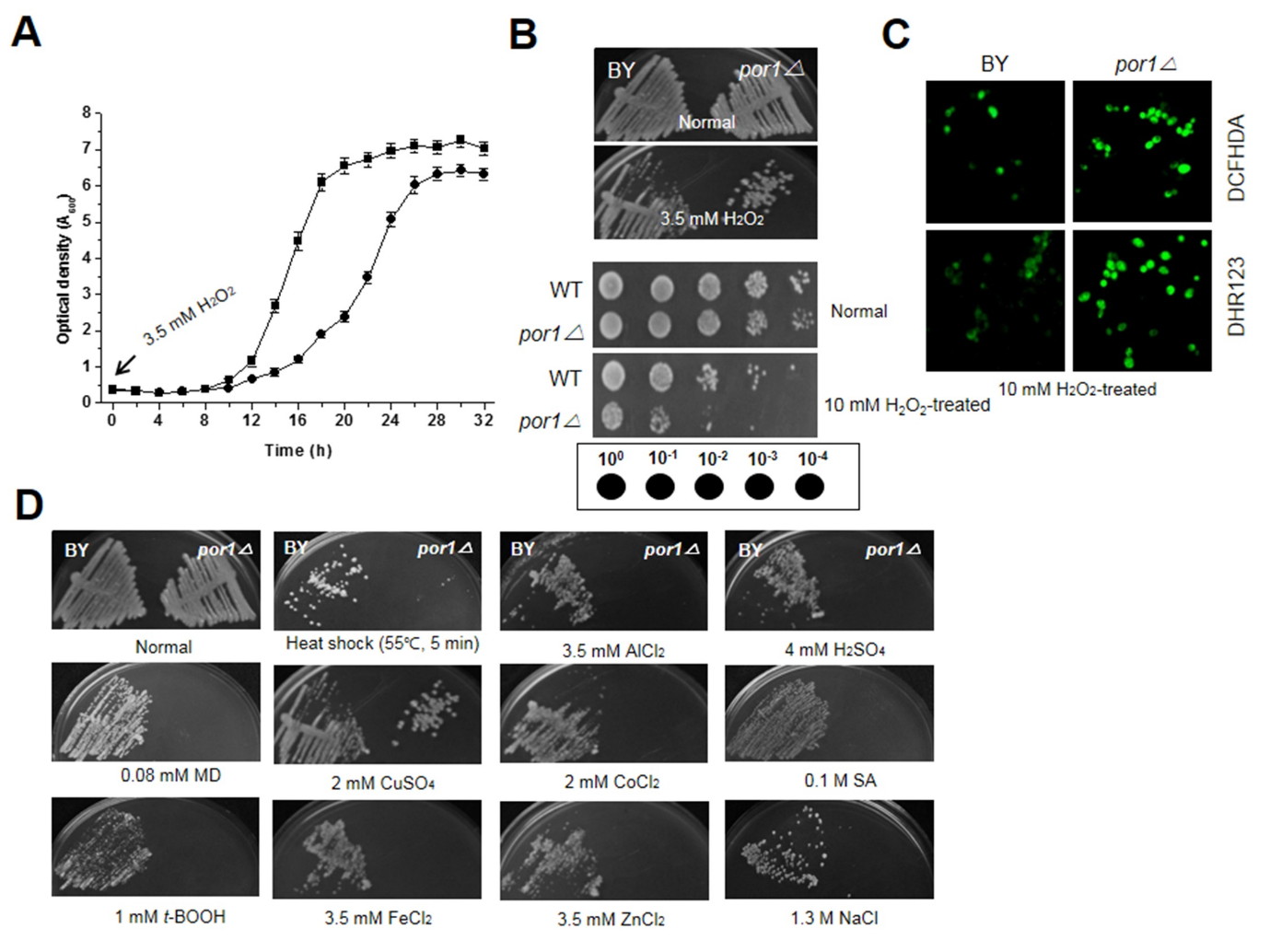
S1 Fig. Stress sensitivity and redox state of *por1△* yeast cells under oxidative stress.** (A) Growth kinetics was observed in YPD medium containing 3.5 mM H_2_O_2_ by monitoring optical density at 600 nm at 2-h intervals for 32 h. Square, BY cells; circle, *por1Δ* cells. (B) Cell survival by streaking (upper panel) and spotting (lower panel) assays in both BY and *por1Δ* cells. For streaking assay, yeast cells were cultured until reaching early log-phase (A_600_ ≈ 1.0) and streaked onto YPD agar plates supplemented with 3.5 mM H_2_O_2_. For spotting assay, yeast cells (A_600_ ≈ 1.0) were exposed to 10 mM H_2_O_2_ for 1 h at 28ºC with shaking, serially diluted to 10^−4^ with YPD medium, and spotted onto YPD agar plates. (C) Cytosolic and mitochondrial redox states were analyzed using the indicator probes for DCFHDA and DHR 123, respectively. Yeast cells were pretreated with the probes for 20 min, washed twice with PBS, and treated with 10 mM H_2_O_2_ for 1 h with shaking. Probe intensity was visualized by fluorescence microscopy. (D) Stress sensitivity of *por1Δ* yeast cells to physiochemical stressors. Yeast cells (A_600_ ≈ 1.0) were streaked onto YPD agar plates containing various stressors as mentioned in *Materials and Methods*. BY, wild-type yeast cells without an empty vector; *por1Δ*, yeast cells in which the *POR1* gene had been deleted.
