# Supplementary material for: Potential Application of the Oryza sativa Monodehydroascorbate Reductase Gene (OsMDHAR) to Improve the Stress Tolerance and Fermentative Capacity of Saccharomyces cerevisiae
Source: PLoS One. 2016 Jul 8;11(7):e0158841. doi: 10.1371/journal.pone.0158841 (PMC4938589; doi:10.1371/journal.pone.0158841)
Supplement: S2 Fig — (DOCX) [file pone.0158841.s002.docx]

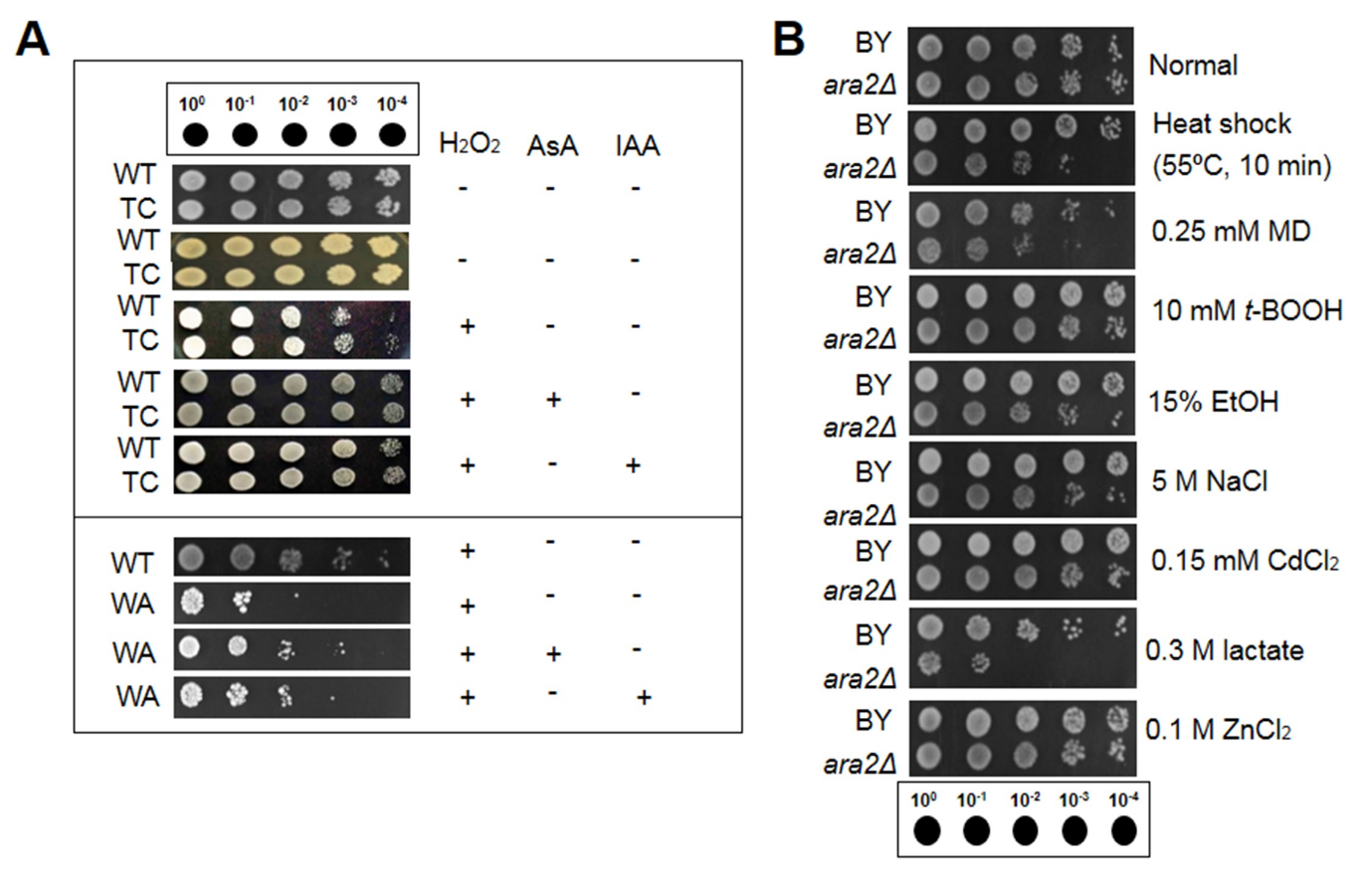


**S2 Fig. Exogenous effect of ascorbate and its analogue on the stress sensitivity of *ara2****Δ* **yeast cells.** (A) Monitoring of the effect was performed by spotting assay. Yeast cells pretreated with 10 mM AsA and 10 mM IAA for 1 h were exposed to 20 mM H_2_O_2_ for 1 h. Five microliters of diluted solutions were spotted onto YPD agar plates. Exogenous AsA- and IAA-treated cells showed better survival compared to cells under normal conditions, suggesting that their concentrations were sufficient for detoxifying endogenous and exogenous ROS and enhanced cell proliferation. WT, yeast cells transformed an empty vector; TC, *OsMDHAR*-expressing yeast cells. (B) Stress sensitivity of *ara2Δ* yeast cells to oxidative stress. Yeast cells were treated with abiotic stressors for 1 h with shaking and spotted onto YPD agar plates. For heat shock, yeast cells were incubated for 5 min at 55ºC, and then spotted as described above. BY, wild-type yeast cells without an empty vector; *ara2Δ*, yeast cells in which the *ARA2* gene had been deleted.
