# Supplementary material for: Potential Application of the Oryza sativa Monodehydroascorbate Reductase Gene (OsMDHAR) to Improve the Stress Tolerance and Fermentative Capacity of Saccharomyces cerevisiae
Source: PLoS One. 2016 Jul 8;11(7):e0158841. doi: 10.1371/journal.pone.0158841 (PMC4938589; doi:10.1371/journal.pone.0158841)
Supplement: S3 Fig — (DOCX) [file pone.0158841.s003.docx]

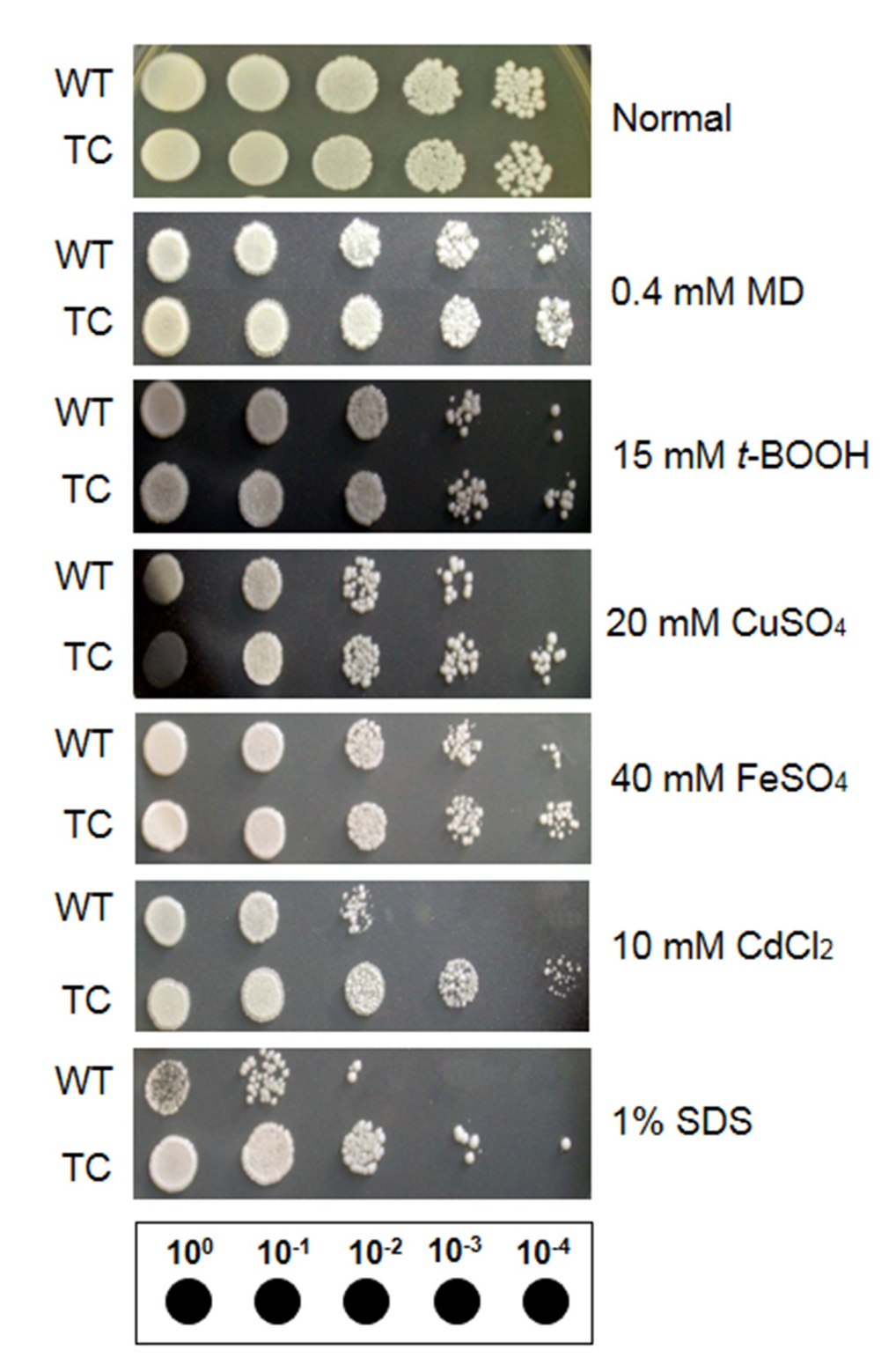


**S3 Fig. Response of *OsMDHAR*-expressing yeast cells to abiotic stressors.** Yeast cells (A_600_ ≈ 2.0) were challenged with various stressors, including 0.4 mM MD, 15 mM *t*-BOOH, 20 mM CuSO_4_, 40 mM FeCl_2_, 10 mM CdCl_2_, and 1% SDS, for 1 h at 28ºC with shaking. Stressed cells were serially diluted to 10^−4^ with YPD medium, spotted onto YPD agar plates, and then incubated for 3 days at 28ºC. WT, yeast cells transformed an empty vector; TC, *OsMDHAR*-expressing yeast cells.
