# Supplementary material for: Potential Application of the Oryza sativa Monodehydroascorbate Reductase Gene (OsMDHAR) to Improve the Stress Tolerance and Fermentative Capacity of Saccharomyces cerevisiae
Source: PLoS One. 2016 Jul 8;11(7):e0158841. doi: 10.1371/journal.pone.0158841 (PMC4938589; doi:10.1371/journal.pone.0158841)
Supplement: S1 Methods — (DOCX) [file pone.0158841.s004.docx]

**Cellular response and redox state in *por1Δ* yeast cells under oxidative stress**

Yeast cells pre-cultured overnight were inoculated (1% of culture volumes) in fresh YPD medium containing 1% yeast extract, 2% peptone, and 2% dextrose, and used for subsequent experiments. For growth kinetics, yeast cells (*por1Δ*) were cultured in YPD medium containing 3.5 mM H_2_O_2_ and monitored by measuring optical density at 600 nm at 2-h intervals for the indicated time. For streaking assay, yeast cells were cultured until reaching early-log phase (A_600_ ≈ 1.0), streaked onto YPD agar (YPD plus 1.5% agar) plates containing various stressors, and incubated for 3 days. Stressors introduced were as follows: 0.08 mM menadione (MD; water-insoluble form), 1 mM *tert*-butylhydroperoxide (*t*-BOOH), 2 mM CuSO_4_, 3.5 mM FeCl_2_, 3.5 mM AlCl_2_, 2 mM CoCl_2_, 3.5 mM ZnCl_2_, 4 mM H_2_SO_4_, 0.1 M salicylic acid (SA), and 1.3 M NaCl. Heat shock was induced for 5 min at 55ºC and then the cells were streaked onto YPD agar plates. Cell viability was also monitored by a spotting assay. Early-log phase yeast cells (A_600_ ≈ 1.0) were treated with 10 mM H_2_O_2_ for 1 h at 28ºC with shaking, and serially diluted with YPD medium. Five microliters were spotted onto YPD agar plates, which were then incubated for 3 days at 28ºC, and photographed. For fluorescence assays, yeast cells were challenged with 0.1 mM DCFHDA and 0.1 mM DHR 123 for 20 min at 28ºC with shaking before 10 mM H_2_O_2_ treatment for 1 h at 28ºC, washed twice with PBS, and visualized by fluorescence microscopy (excitation, 488 nm; emission, 525 nm).
