# Supplementary material for: Potential Application of the Oryza sativa Monodehydroascorbate Reductase Gene (OsMDHAR) to Improve the Stress Tolerance and Fermentative Capacity of Saccharomyces cerevisiae
Source: PLoS One. 2016 Jul 8;11(7):e0158841. doi: 10.1371/journal.pone.0158841 (PMC4938589; doi:10.1371/journal.pone.0158841)
Supplement: S2 Methods — (DOCX) [file pone.0158841.s005.docx]

**Cellular response and redox state in *ara2△* yeast cells** **under oxidative stress and exogenous effect of AsA and its analogue**

To investigate the stress sensitivity of *ara2△*, yeast cells (A_600_ ≈ 1.0) were exposed to abiotic stressors including 0.25 mM MD, 10 mM *t*-BOOH, 15 % ethanol, 5 M NaCl, 0.15 mM CdCl_2_, 0.3 M lactic acid, and 0.1 M ZnCl_2_ for 1 h at 28ºC. Stressed yeast cells were diluted to 10^−4^ with YPD medium, spotted onto YPD agar plates and then incubated for 3 days at 28ºC. Mid-log phase yeast cells (A_600_ ≈ 2.0) were pretreated with 10 mM AsA and 10 mM IAA for 1 h at 28ºC with shaking, washed twice with YPD medium to remove residual AsA and IAA, treated with 20 mM H_2_O_2_ for 1 h at 28ºC with shaking, and then serially diluted with YPD medium. Five microliters of the diluted solutions were spotted onto YPD agar plates.
