# Supplementary material for: Potential Application of the Oryza sativa Monodehydroascorbate Reductase Gene (OsMDHAR) to Improve the Stress Tolerance and Fermentative Capacity of Saccharomyces cerevisiae
Source: PLoS One. 2016 Jul 8;11(7):e0158841. doi: 10.1371/journal.pone.0158841 (PMC4938589; doi:10.1371/journal.pone.0158841)
Supplement: S3 Methods — (DOCX) [file pone.0158841.s006.docx]

**Cellular response and redox state in yeast under oxidative stress**

To monitor the acquired tolerance of *OsMDHAR-*expressing cells, yeast cells (A_600_ ≈ 2.0) were challenged with abiotic stresses, including 0.4 mM MD, 15 mM *t*-BOOH, 20 mM CuSO_4_, 40 mM FeSO_4_, 10 mM CdCl_2_, and 1% sodium dodecyl sulfate (SDS) for 1 h at 28ºC. Stressed yeast cells were diluted to 10^−4^ with YPD medium, spotted onto YPD agar plates, incubated for 3 days at 28ºC, and then photographed.
