# Supplementary material for: Potential Application of the Oryza sativa Monodehydroascorbate Reductase Gene (OsMDHAR) to Improve the Stress Tolerance and Fermentative Capacity of Saccharomyces cerevisiae
Source: PLoS One. 2016 Jul 8;11(7):e0158841. doi: 10.1371/journal.pone.0158841 (PMC4938589; doi:10.1371/journal.pone.0158841)
Supplement: S1 Table — (DOCX) [file pone.0158841.s007.docx]

**S1 Table. Oligonucleotide sequence used in this study.**

| Oligo name | Oligonucleotide sequence (5´-3´) |
| --- | --- |
| MDHAR-NcoI-F | GCGGCAG**CCATGG**CGTCGGAGAAGCACTTCAAG |
| MDHAR-KpnI-R | GTAGTA**GGTACC**TTCTTTATTCAAATCTCAGCAGC |
| OsMDHAR-F | CGATGGTGTTTCCTGAACCT |
| OsMDHAR-R | GTCGAATGACCGGGAGTAGA |
| ARA2-F | CGTATTGGTGCGGAAGAGTT |
| ARA2-R | TCATGAGAACACGGGTGAAA |
| PDA1-F | CTTCATTCAAACGCCAACCA |
| PDA1-R | GAGGCAAAACCTTGCTTTTTG |

*****Restriction enzyme sites were underlined in bold form.
