# Supplementary material for: Potential Application of the Oryza sativa Monodehydroascorbate Reductase Gene (OsMDHAR) to Improve the Stress Tolerance and Fermentative Capacity of Saccharomyces cerevisiae
Source: PLoS One. 2016 Jul 8;11(7):e0158841. doi: 10.1371/journal.pone.0158841 (PMC4938589; doi:10.1371/journal.pone.0158841)
Supplement: S2 Table — (DOCX) [file pone.0158841.s008.docx]

**S2 Table. Strains and plasmids used in this study.**

| Host, strain and plasmid | Genotype and description | Source |
| --- | --- | --- |
| Rice (*Oryza sativa*) | | |
| WT | *Oryza sativa* L. *japonica* “Ilmi” | This study |
| *Saccharomyces cerevisiae* | | |
| BY4741(BY) | *MATa; his3Δ1; leu2Δ0; met15Δ0; ura3Δ0* | Euroscarf |
| *ara2Δ*  *por1Δ*  *por2Δ*  *sod1Δ*  *tsa1Δ* | *MATa; his3Δ1; leu2Δ0; met15Δ0; ura3Δ0; YMR041c::kanMX4*  *MATa; his3Δ1; leu2Δ0; met15Δ0; ura3Δ0;* *YNL055c::kanMX4*  *MATa; his3Δ1; leu2Δ0; met15Δ0; ura3Δ0;* *YIL114c::kanMX4*  *MATa; his3Δ1; leu2Δ0; met15Δ0; ura3Δ0;* *YJR104c::kanMX4*  *MATa; his3Δ1; leu2Δ0; met15Δ0; ura3Δ0;* *YML028w::kanMX4* | ATCC  ATCC  ATCC  ATCC  ATCC |
| WT | *MATa; his3Δ1; leu2Δ0; met15Δ0; ura3Δ0; p426GPD* | This study |
| TC  WA  TA | *MATa; his3Δ1; leu2Δ0; met15Δ0; ura3Δ0; p426GPD::OsMDHAR*  *MATa; his3Δ1; leu2Δ0; met15Δ0; ura3Δ0; YMR041c::kanMX4; p426GPD*  *MATa; his3Δ1; leu2Δ0; met15Δ0; ura3Δ0; YMR041c::kanMX4; p426GPD::OsMDHAR* | This study  This study  This study |
| Plasmid | | |
| p426GPD | *S. cerevisiae* expression vector | Euroscarf |
| p426GPD::OsMDHAR | *OsMDHAR* expression vector in *S. cerevisiae* | This study |

*Euroscarf, European Saccharomyces cerevisiae Archive for Functional Analysis; ATCC, American Type Culture Collection.
